# Functional Mechanism of C-Terminal Tail in the Enzymatic Role of Porcine Testicular Carbonyl Reductase: A Combined Experiment and Molecular Dynamics Simulation Study of the C-Terminal Tail in the Enzymatic Role of PTCR

**DOI:** 10.1371/journal.pone.0090712

**Published:** 2014-03-19

**Authors:** Minky Son, Woo Young Bang, Chanin Park, Yuno Lee, Seul Gi Kwon, Sam Woong Kim, Chul Wook Kim, Keun Woo Lee

**Affiliations:** 1 Division of Applied Life Science (BK21 Plus Program), Systems and Synthetic Agrobiotech Center (SSAC), Plant Molecular Biology and Biotechnology Research Center (PMBBRC), Research Institute of Natural Science (RINS), Gyeongsang National University (GNU), 501 Jinju-daero, Gazha-dong, Jinju, Republic of Korea; 2 Industry-Academic Cooperation Foundation, Gyeongnam National University of Science & Technology, Jinju, Republic of Korea; 3 Swine Science and Technology Center, Gyeongnam National University of Science & Technology, Jinju, Republic of Korea; Oak Ridge National Laboratory, United States of America

## Abstract

Porcine testicular carbonyl reductase, PTCR which is one of the short chain dehydrogenases/reductases (SDR) superfamily catalyzes the NADPH-dependent reduction of carbonyl compounds including steroids and prostaglandins. Previously we reported C- terminal tail of PTCR was deleted due to a nonsynonymous single nucleotide variation (nsSNV). Here we identified from kinetic studies that the enzymatic properties for 5α-dihydrotestosterone (5α-DHT) were different between wild-type and C-terminal-deleted PTCRs. Compared to wild-type PTCR, C-terminal-deleted PTCR has much higher reduction rate. To investigate structural difference between wild-type and C-terminal-deleted PTCRs upon 5α-DHT binding, we performed molecular dynamics simulations for two complexes. Using trajectories, molecular interactions including hydrogen bonding patterns, distance between 5α-DHT and catalytic Tyr193, and interaction energies are analyzed and compared. During the MD simulation time, the dynamic behavior of C-terminal tail in wild-type PTCR is also examined using essential dynamics analysis. The results of our simulations reveal that the binding conformation of 5α-DHT in C-terminal-deleted PTCR is more favorable for reduction reaction in PTCR, which shows strong agreement with kinetic data. These structural findings provide valuable information to understand substrate specificity of PTCR and further kinetic properties of enzymes belonging to the SDR superfamily.

## Introduction

Porcine testicular carbonyl reductase, PTCR (also known as 20β-hydroxysteroid dehydrogenase, 20β-HSD) belongs to the short-chain dehydrogenases/reductases (SDR) superfamily. The SDR catalyze crucial steps of activation and inactivation of steroids, vitamins, prostaglandins, and other bioactive molecules by oxidation and reduction of hydroxyl and carbonyl groups, respectively [1]. Almost all SDRs have Tyr-Lys-Ser as catalytic triad and their functional units are homotetramers or homodimers [1]–[8]. However, interestingly, PTCR is the first known monomeric structure among the SDR superfamily and it catalyzes the NADPH-dependent reduction of ketones on androgens, aldehydes, progestins, and prostaglandins as well as various xenobiotics [9], [10]. The high activity of enzyme is shown by the reduction of 20-carbonyl groups of C_21_-steroids, such as conversion of 17α-hydroxyprogesterone to 17α, 20β-dihydroxy-4-pregnen-3-one, which is present in pig testes during the neonatal period [11], [12]. Purified PTCR has enzymatic activities to 3α-hydroxysteroid, 3β-hydroxysteroid and 5α-dihydrotestosterone (5α-DHT) as substrates [13]. Meantime, the endogenous PTCR proteins were identified as two bands, 31 kDa and 30 kDa proteins from porcine testis through western blot analysis even though the reason for detection of a minor 30 kDa protein remained unclear [14]. Recently, our report described that the minor 30 kDa protein, detected in porcine testis, is a C-terminal-deleted PTCR, expressed from the PTCR gene having a paralogous sequence variant (T), probably generated by gene duplication [15]. It also suggested that the nonsynonymous variation (G>T), generated by gene duplication, leads to a deletion of the C-terminal region (E281 to A288) of PTCR, strongly indicating that the pig testicular genome endogenously produces both wild-type and C-terminal-deleted PTCR, 31 kDa and 30 kDa proteins, respectively [15]. Additionally, the C-terminal fragment from E281 to A288, in the PTCR structure resolved at high resolution, is reported to be in the vicinity of the active site [16] and is absent in human carbonyl reductase despite the high homology (about 85%) between PTCR and human carbonyl reductase [9], [16]. Accordingly, it has been suggested that the PTCR-unique C terminus might provide a clue to the differential kinetics between porcine and human carbonyl reductases [9], and the structural importance of the C-terminal tail also has been discussed with regard to catalysis of the PTCR enzyme [17].

In the present study, we found that WT and C-terminal-deleted PTCRs have shown different kinetics in NADPH-dependent carbonyl reductase activity. To find structure related mechanistic explanation for the different enzymatic properties between WT and C-terminal-deleted PTCRs, molecular dynamics (MD) simulations were performed. Using trajectories, molecular interactions including hydrogen bonding patterns, distance between and catalytic Tyr193 and interaction energies are analyzed and compared. During the MD simulation time, the dynamic behavior of C-terminal tail in WT PTCR is also examined using essential dynamics analysis. Our simulation results provide detailed information which can explain the different binding mode of 5α-DHT in WT and C-terminal-deleted PTCRs and these explanations are in the agreement with the kinetic experimental data.

## Materials and Methods

### Materials

The following chemicals were used in the experiments: Na_2_HPO_4_, NaH_2_PO_4_, NaCl, bovine serum albumin (BSA), imidazole, 5α-DHT, methylglyoxal, 9,10-phenanthrenequinone and hydrindantin were purchased from Sigma (St. Louis, MO), and Ni-NTA chelating agarose CL-6B was purchased from Peptron company (Daejon, Korea). Bradford Protein assay kit and Ultrafree-0.5 Centrifugal Filter Device were purchased from Bio-Rad (Richmond, CA) and Millipore (Bedford, MA), respectively.

### Recombinant protein purification

Two types of pPROEX HTb-PTCR clones were already constructed in previous study [1] and further used for the IPTG-induced expression of each of the clones in *E. coli* BL21, in order to produce the his-tagged fusion proteins, PTCR(WT) and PTCR(ΔCterm). Subsequently, the IPTG-induced proteins were subjected to the affinity chromatography using Ni-NTA agarose, according to manufacturer's manual (Peptron, Daejon, Korea). Briefly, basal buffer for protein purification was prepared by 50 mM sodium phosphate buffer (pH 8.0) and 500 mM NaCl, and imidazole (Sigma, USA) was added by the concentrations required for the affinity chromatography. The overexpressed cells was precipitated by centrifugation and suspended by binding buffer, the basal buffer including 5 mM imidazole. The genomic DNAs of collected cells were fragmented by SONICS Vibracell VCX 750 Ultrasonic Cell Disruptor (Newtown, CT), which was done twice by conditions as following; 5 min by 2 sec interval of on/off and 35% amplitude during ice cooling. The supernatant to obtain water-soluble protein was collected from the centrifugation at 12,000× g for 30 min at 4°C, afterward the supernatant was loaded to a column including the Ni-NTA agarose, washed by the above binding buffer and then subjected to the stepwise elution using the basal buffer including various imidazole concentrations. The purified recombinant proteins were concentrated by Ultrafree-0.5 Centrifugal Filter Device (Millipore, Bedford, MA) and then were dissolved with 50% glycerol and 50 mM Sodium Phosphate Buffer (pH 6.4) for long-term storage at −20°C. Finally, concentrations of the recombinant proteins, PTCR(WT) and PTCR(ΔCterm), were determined by Bradford Protein assay kit (Bio-Rad, Richmond, CA) as about 4.57 µg/µl and 1.26 µg/µl, respectively.

### Measurement of NADPH-dependent carbonyl reductase activity

**Figure 1:**
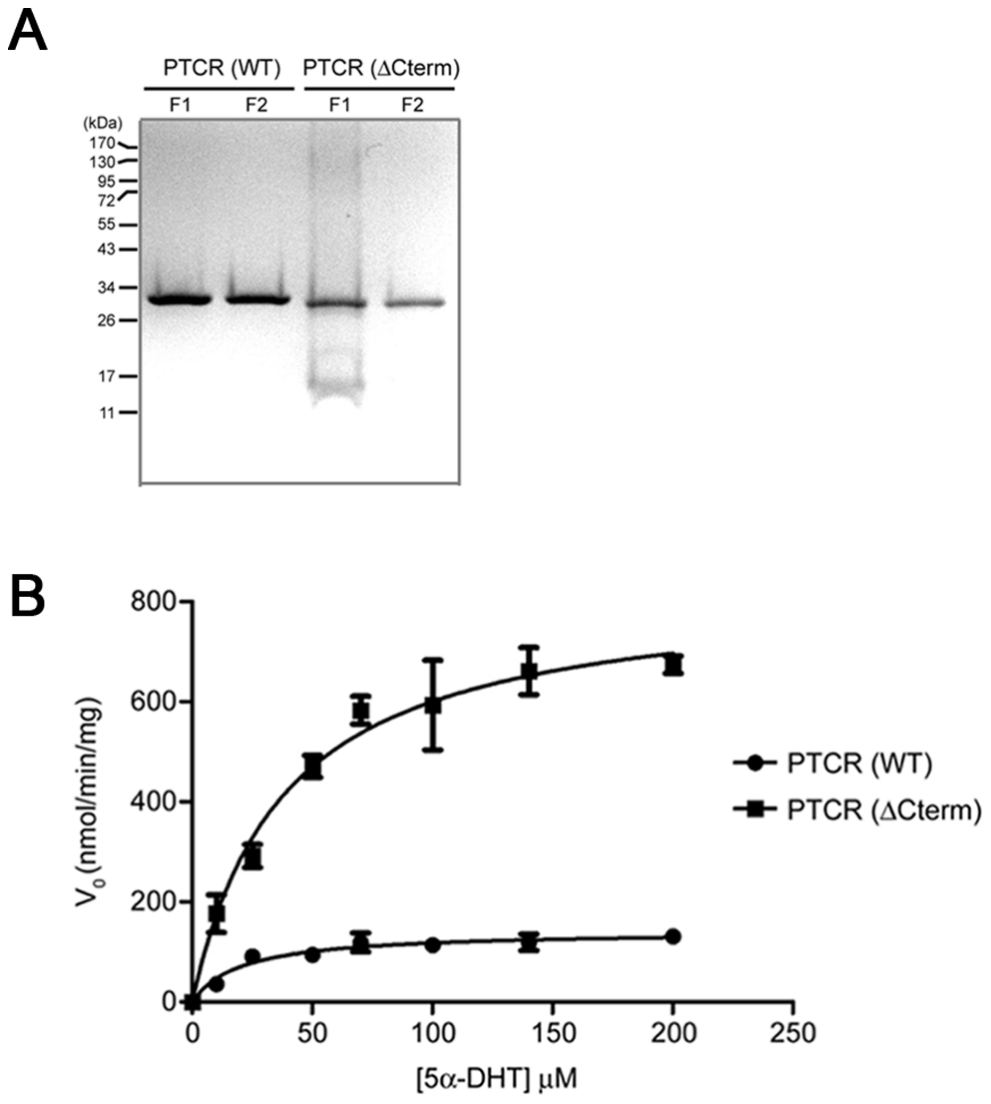
A Michaelis-Menten plot from measurement of the NADPH-dependent reduction of 5α-DHT by recombinant PTCR(WT) or PTCR(ΔCterm). (A) Purification of his-tagged recombinant proteins, PTCR(WT) and PTCR(ΔCterm). After stepwise elution with various imidazole concentrations, each fraction was loaded onto 12% SDS-polyacrylamide gels. The F indicates a fraction. (B) Michaelis-Menten plot. The data, obtained from measurement of the NADPH-dependent reduction of 5α-DHT by recombinant PTCR(WT) and PTCR(ΔCterm), were analyzed by nonlinear regression using GraphPad prism 6 software (GraphPad Software Inc., San Diego, CA). Each spot represents the mean ± S.E. (n = 3).

**Figure 2:**
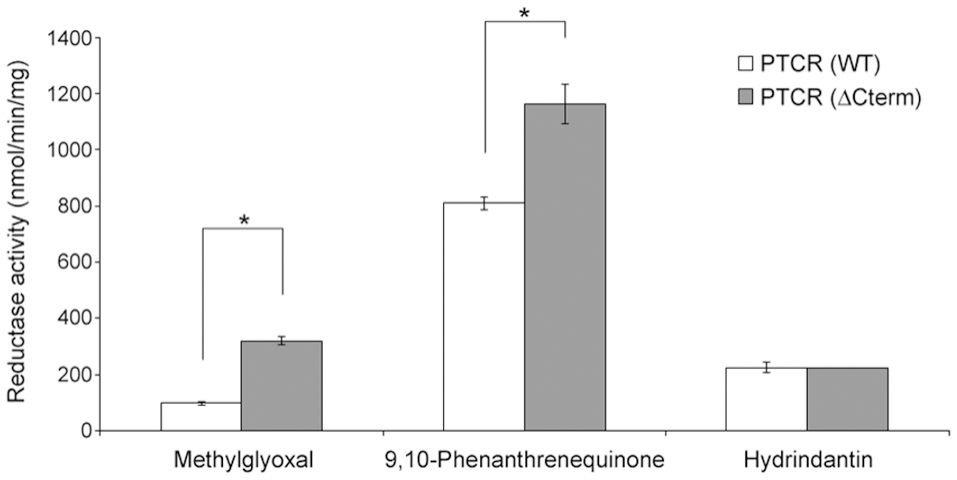
Comparison of the NADPH-dependent reduction activities of carbonyl compounds by the PTCR (WT) and the PTCR (ΔCterm). In addition to the 5α-DHT, other carbonyl compounds such as 50 µM methylglyoxal, 100 µM 9,10-phenanthrenequinone and 100 µM hydrindantin were used as substrates for the analysis of NADPH-dependent carbonyl reductase activities by the PTCR (WT) and the PTCR (ΔCterm). The bar indicates the mean ± S.E. (n = 3) and the * represent a significant difference (*p*<0.01), analyzed by the Student's t-test.

Reaction mixtures consisted of 60 mM sodium phosphate (pH 6.5), the purified PTCR(WT) or PTCR(ΔCterm) proteins, 0.1 mM NADPH, and various amounts of substrates as indicated in Figure 1 and 2 were incubated in a quartz cuvette at 37°C. The assays of NADPH-dependent reduction activity for various substrates were carried out spectrophotometrically by monitoring the change in absorbance at 340 nm with time.

### Statistical analysis

To determine kinetic parameters with a Michaelis–Menten plot, the data were analyzed by nonlinear regression using GraphPad prism 6 software (GraphPad Software Inc., San Diego, CA). Et values, 1711 and 1759, were used for the calculation of turnover rates (*K_cat_*) for PTCR(WT) and PTCR(ΔCterm), respectively. The significant differences were analyzed by Student's t-test (*p*<0.01 or *p*<0.05) using the above software. Results are expressed as means ± standard errors (S.E.) of at least 3 independent experiments.

### Preparation of PTCR complex structure

The WT PTCR and C-terminal (E281 to A288)-deleted PTCR that each protein in complex with NADPH and 5α-DHT were already modeled through molecular docking simulation in our previous study [15]. The binding conformations of 5α-DHT at the active site of WT and C-terminal-deleted PTCRs were predicted using GOLD program [18], [19]. The substrate binding site was defined by 20 Å around the center of the site between C-terminal tail and NADPH. During the calculation, the number of GA runs was set to 30 and the predicted poses were ranked by GOLD fitness score. All other parameters were used as their defaults.

### Molecular dynamics simulations

Two modeled structures were then used as a starting structure for molecular dynamics simulation. The MD simulations were carried out with CHARMM27 force field using GROMACS 4.5.3 package [20], [21]. The topology files for cofactors and substrates were generated using SwissParam [22]. As a validation purpose, we calculated the topologies of cofactors and substrates using ParamChem [23]–[25] and MATCH [26] and compared with the topologies obtained from SwissParam. At first, hydrogen atoms were added and all the ionizable residues in the protein were protonated at pH 7. A cubic water box of 1.5 nm from the surface of the protein was generated and solvated with TIP3P water model [27] to perform the simulations in an aqueous environment. The Na^+^ counter-ions were added by replacing water molecules to neutralize the system. The systems were subjected to energy minimization using steepest descent algorithm to improve the model quality to convergence on maximum force lower than 2000 kJ/mol. The energy minimized systems were then equilibrated in three steps. In the first step, NVT equilibration was conducted for 100 ps at 300 K. A constant temperature was controlled by V-rescale thermostat [28]. After NVT, 100 ps NPT ensemble was applied at 1 bar of pressure followed by 20 ns production run under the same ensemble. During this process, 300 K and 1 bar was kept using the same thermostat and Parrinello-Rahman barostat [29], [30]. For the equilibration process, the protein backbone was restrained and the solvent molecules with counter-ions were allowed to move. Bonds between heavy atoms and corresponding hydrogen atoms were restrained to their equilibrium bond lengths using the LINCS algorithm [31], [32] and the geometry of water molecules was constrained using SETTLE algorithm [33]. Short-range interactions were considered by using the cut-off value of 1.2 nm and long-range electrostatic interaction was calculated using particle mesh Ewald (PME) method [34], [35]. A grid spacing of 0.12 nm was applied for fast Fourier transform calculations. All simulations were performed under periodic boundary conditions to avoid edge effects. The time step of the simulation was 2 fs and the coordinate data were stored to the file every 1 ps. We repeated the simulation of each system four times (Rep 1 to Rep 4) under the same conditions. All analysis was done using VMD, Discovery studio (DS) v3.1, and GROMACS. The representative structure which is the closest conformation to the average structure was selected from each simulation and used for the analysis. Essential dynamics analysis [36], [37] also called principal component analysis was performed. The covariance matrices were diagonalized by constructing projections of trajectories on the eigenvectors. Movements of all C_α_ atoms of the protein in the essential subspace were projected according to the most significant eigenvectors.

## Results and Discussion

### The C-Terminal Region Contributes Significantly to the Enzymatic Properties of PTCR

Previously, the PTCR had been identified as two differential variants with 31 kDa or 30 kDa in porcine testis [9]. Recently, our approach integrating RNA-Seq and molecular biological analyses supported that the minor 30 kDa protein, detected in porcine testis, is a C-terminal (E281 to A288)-deleted PTCR, expressed from the *PTCR* gene having a paralogous sequence variant [15]. In addition, the C-terminal region (E281 to A288) was suggested to contribute to different kinetic properties between the NADPH-dependent carbonyl reductase activities of WT and C-terminal-deleted PTCRs [15]. Thus, these indicate that the C-terminal region (E281 to A288) of PTCR has a significant effect on catalytic turnover rate (*k*_cat_) with a slight one on substrate binding affinity (*K*_m_).

To identify their kinetic characteristics, the his-tagged PTCR(WT) and PTCR(ΔCterm), recombinant PTCR proteins with or without the C-terminal region, were purified by affinity chromatography using a Ni-NTA resin. After stepwise elution with imidazole, the results of SDS-PAGE revealed that several fractions contain the high purity proteins such as his-tagged (about 1 kD) PTCR (WT) and PTCR (ΔCterm), which have different protein sizes of about 32 kD and 31 kDa, respectively (Figure 1A). Subsequently, the highly purified proteins were subjected to the investigation of NADPH-dependent carbonyl reductase activity using 5α-DHT as a substrate. As shown in the Michaelis–Menten plot (Figure 1B), the rates of 5α-DHT reduction by PTCR (WT) and PTCR (ΔCterm) were significantly different: PTCR(ΔCterm) showed much higher reduction rate than PTCR(WT) did. In addition, the kinetic parameters, determined from the Michaelis–Menten plot (Figure 1B), revealed significant increases in *V*_max_ (*p*<0.01), *k*_cat_ (*p*<0.01), *K*_m_ (*p*<0.05), and *k*_cat_/*K*_m_ (*p*<0.01) for the NADPH-dependent 5α-DHT reduction by the PTCR(ΔCterm), compared to the PTCR(WT) (Table 1). Approximately a 5-fold increase was observed in the 5α-DHT reduction rate (*V*_max_ or *k*_cat_) by the PTCR(ΔCterm), with a slight increase in the *K*_m_ for this reaction, compared to the PTCR(WT). Therefore, the catalytic efficiency (*k*_cat_/*K*_m_) for the 5α-DHT reduction increased by *V*_max_ when comparing the PTCR(ΔCterm) to the PTCR(WT), primarily as a result of a markedly increased *V*_max_ (or *k*_cat_) despite of a slightly increased *K*_m_ for 5α-DHT reduction by the PTCR(ΔCterm) (Table 1). Besides, the comparison of their NADPH-dependent carbonyl reductase activities were assessed using other carbonyl compounds such as methylglyoxal, 9,10-phenanthrenequinone and hydrindantin as substrates. The PTCR(ΔCterm) showed significantly higher reduction rates than the PTCR(WT) when using the methylglyoxal and 9,10-phenanthrenequinone as substrates, like using the 5α-DHT as a substrate, whereas it showed no difference when using the hydrindantin as a substrate (Figure 2), which suggests that the C-terminal region (E281 to A288) has a significant effect on substrate specificity of PTCR. Altogether, our results indicate that the C-terminal region (E281 to A288) contributes to the different enzymatic properties, such as the catalytic turnover rate, the substrate binding affinity and even the substrate specificity, between PTCR(WT) and PTCR(ΔCterm). Furthermore, these imply that the PTCR may have evolved its C-terminal region by generating a paralogous sequence variant through gene duplication.

**Table 1:** Kinetic parameters of reduction of 5α-DHT with PTCR (WT) or PTCR (ΔCterm).

	Kinetic Parameters			
	*V*_max_ (nmol/min/mg)	*K*_m_ (µM)	*k*_cat_ (s^−1^)	*k*_cat_/*K*_m_ (s^−1^ M^−1^)
PTCR(WT)	142.2±9.80	20.31±1.905	0.08312±0.005730	4,116±139.7
PTCR(ΔCterm)	830.6±46.69	38.15±4.125	0.4722±0.02653	12,512±629.3
*P* value^a^	0.0001	0.0172	0.0001	0.0002

Each value indicates mean ± SEM (n = 3).

Kinetic parameters were determined using data in Figure 1B through GraphPad prism 6 software (GraphPad Software Inc., San Diego, CA).

a Student's t-test was used to verify significant differences of kinetic parameters between the reduction activities of 5α-DHT by the PTCR (WT) and the PTCR (ΔCterm).

### MD Simulation of WT and C-terminal-deleted PTCRs in Complex with Its Substrate, 5α-DHT

**Figure 3:**
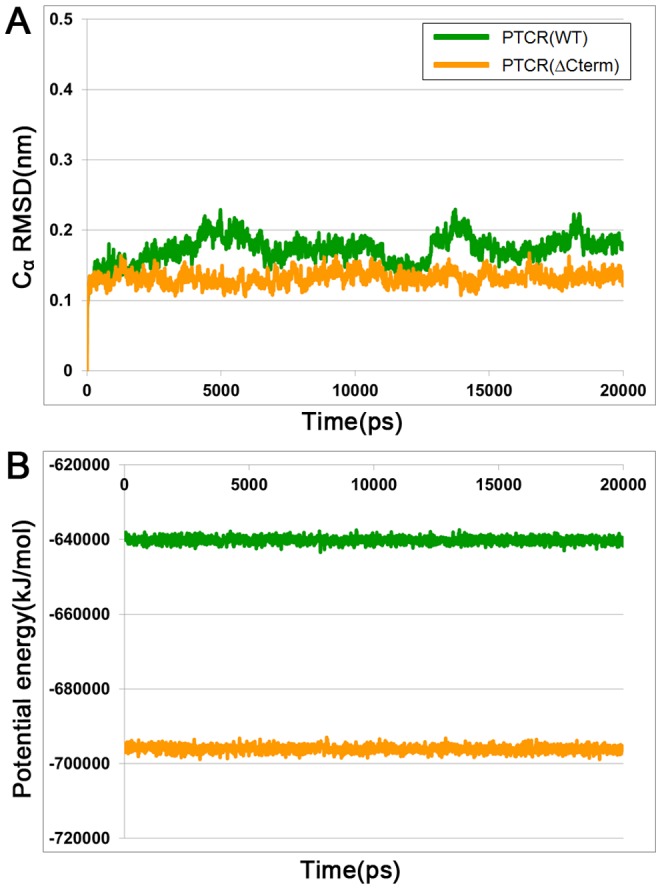
Overall stability of 20 ns MD simulation for WT and C-terminal-deleted PTCRs. (A) The root-mean-square deviation (RMSD) for C_α_ atoms of the protein (B) Potential energy for each system. Both were calculated during the MD simulation time. WT and C-terminal-deleted PTCRs are colored in green and yellow lines, respectively.

To investigate structural difference between WT and C-terminal-deleted PTCRs upon 5α-DHT binding, 20 ns MD simulations were performed using the docked conformations as initial structures. The detail information about the system used in MD simulations is listed in Table 2. During the simulation time, the root-mean-square deviation (RMSD) for the C_α_ atoms of protein and potential energy were measured to examine the stability of the system. The RMSD values for two systems were calculated with respect to their initial configurations, as a function of the simulation time. Both simulations achieve stability in the atom positional RMSD with values less than 0.23 nm (Figure 3A). The RMSD value of WT PTCR is gradually increased up to 0.22 nm until 5000 ps and then is stabilized for the remainder of the 20 ns. Whereas, the RMSD values of C-terminal-deleted PTCR is increased up to 0.13 nm at the first 90 ps of the simulation. After this time, the RMSD are remained stable until the end of the simulation time. The average RMSDs of each system during the last 5 ns are 0.18 and 0.13 nm, respectively. The potential energy calculation also reflects the stability of the both systems (Figure 3B). The energy of each system maintains a constant as average values of −6.40×10^5^ kJ/mol for WT PTCR and −6.96×10^5^ kJ/mol for C-terminal-deleted PTCR during the simulation time. From these results, two systems are stable and there are no abnormal conformational changes in the proteins throughout the 20 ns simulation time. During the last 5 ns of the simulation, the snapshot which is closest conformation to the average structure is selected as representative structure of each system for structural comparison between WT and C-terminal-deleted PTCRs.

**Table 2:** The details of two systems used in molecular dynamics simulations study.

System	No. of atoms	No. of water molecules	No. of counter-ions	System size (nm)
Wild-type PTCR+NADPH+5α-DHT	4583	15056	3	4.79×5.62×4.72
C-terminal (E281-A288)-deleted PTCR+NADPH+5α-DHT	4458	16473	2	4.82×5.79×4.19

### Binding Mode of 5α-DHT at the Active Site of WT and C-terminal-deleted PTCRs

**Figure 4:**
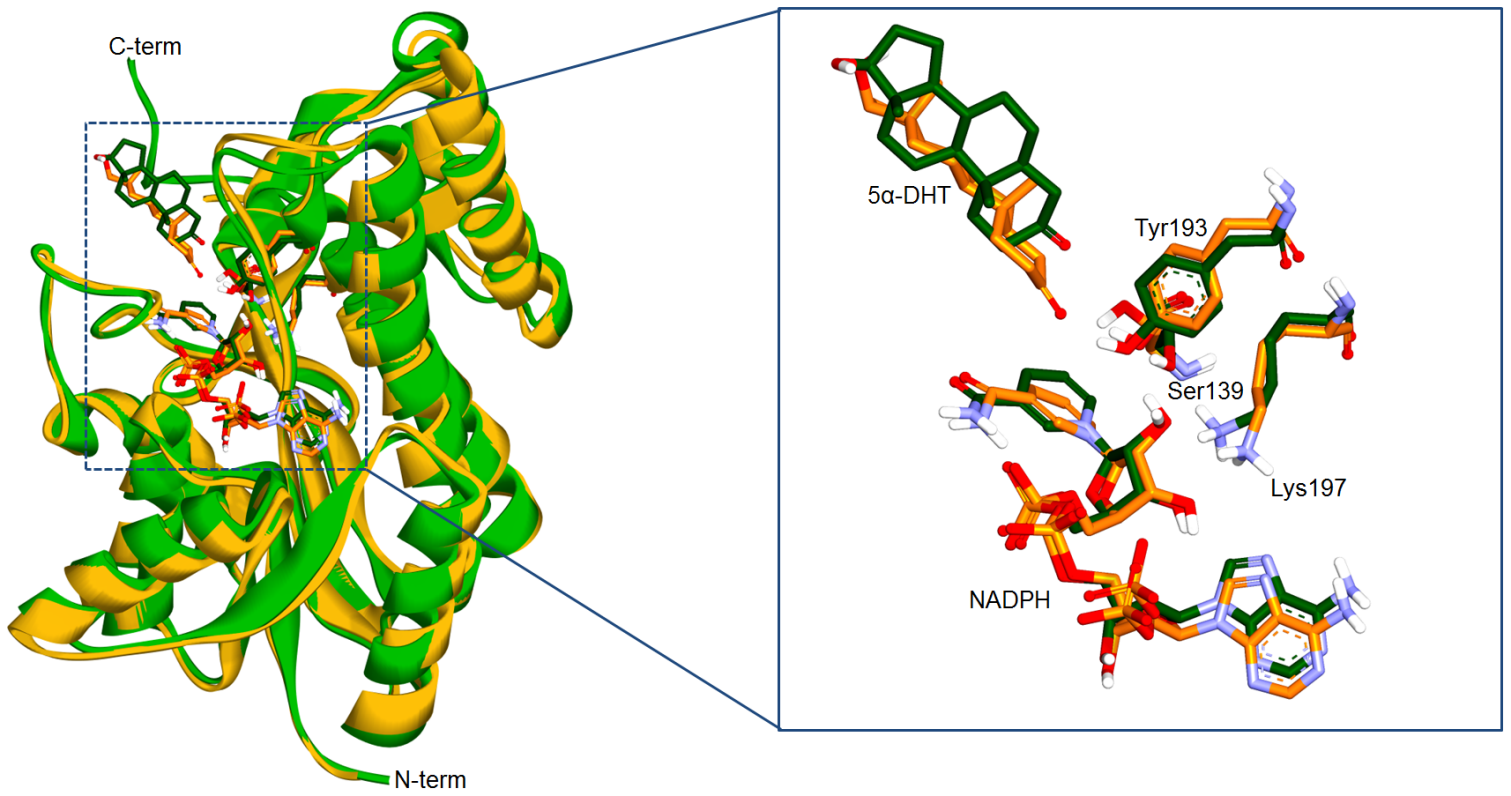
Superposition between representative structures of WT and C-terminal-deleted PTCRs. Two structures are superimposed on C_α_ atoms of the proteins (left). WT PTCR (green) and C-terminal-deleted PTCR (yellow) are shown as cartoon models. The active site is enlarged for a clear view (right) and catalytic triad, 5α-DHT, and NADPH are labeled and represented as stick models.

The binding modes of 5α-DHT in WT and C-terminal-deleted PTCRs are analyzed and compared using their representative structures to understand structural differences induced by the substrate binding. Superimposition of WT PTCR with C-terminal-deleted PTCR showed that there was no significant difference with C_α_ RMSDs of 0.94 Å. The catalytic triad (Ser139, Tyr193, and Lys197) and cofactor (NADPH) are well conserved in the active site in both structures (Figure 4). In case of the NADPH binding, it reveals similar conformations in the both structures, which is consistent with typical cofactor binding in the SDRs to facilitate hydride transfer reaction catalyzed by enzyme [2], [4]–[7], [38], [39]. The C4 atom of the nicotinamide ring, specifically 4-pro-S hydride of NADPH, is facing towards to the carbonyl group of 5α-DHT. Hydrogen bonds between NADPH and active site residues such as Asn13, Lys14, Ile16, Gly17 Arg37, Arg41, Ile63, Asn89, Tyr193, Lys197, Val230, Thr232, and Met234 were observed and the residues including Ile92, Val137, Ser138, Thr140, Cys226, Pro227, Gly228, and Trp229 were involved in hydrophobic interactions (data not shown).

**Figure 5:**
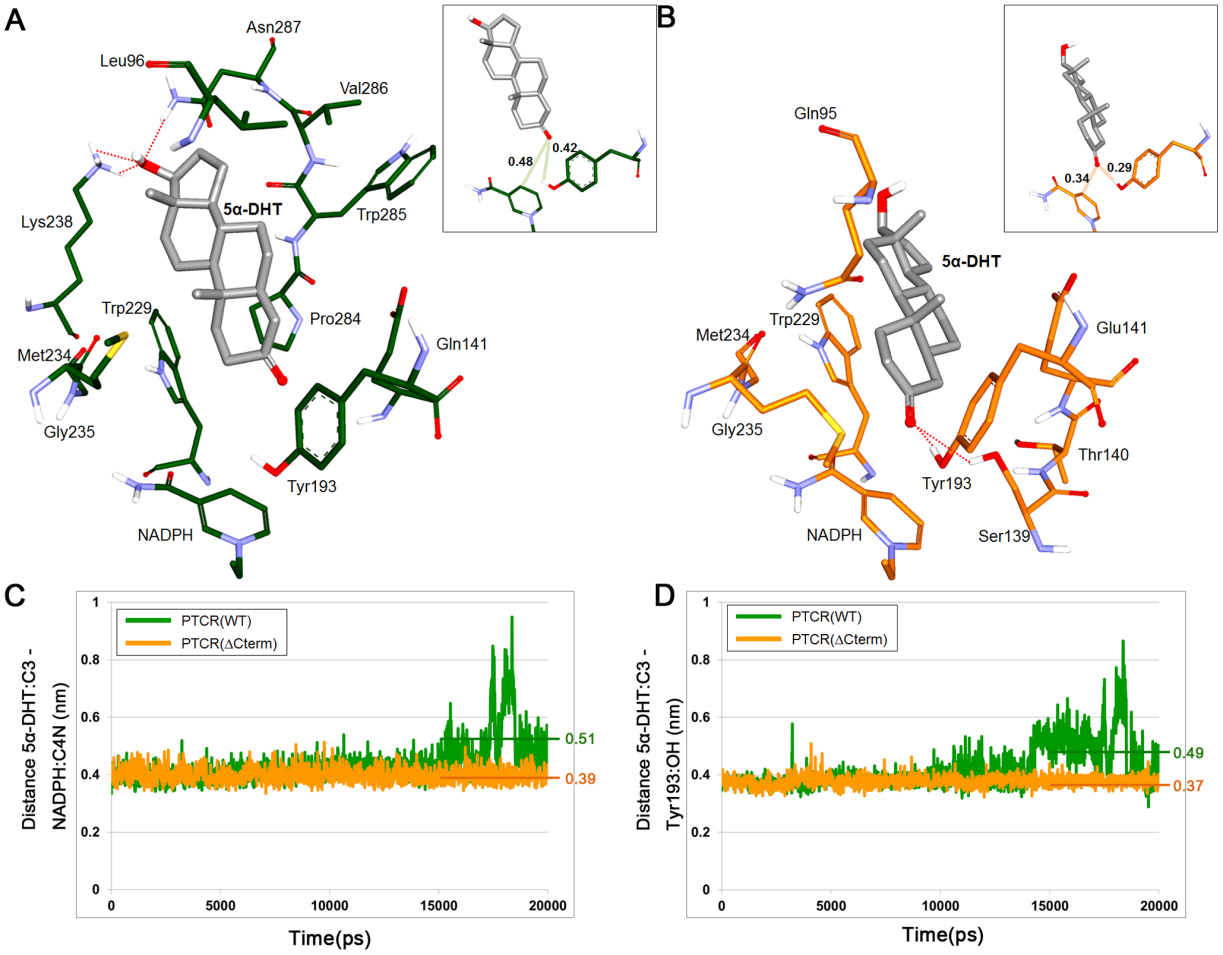
The binding mode of 5α-DHT in the representative structure of WT and C-terminal-deleted PTCRs and comparison of the important distance. The binding conformation of 5α-DHT in the active site of WT PTCR (A) and C-terminal-deleted PTCR (B) were analyzed. Interacting residues in WT PTCR and C-terminal-deleted PTCR are displayed as green and yellow stick models. The polar hydrogen atoms are shown and 5α-DHT is drawn as gray stick model in the both structures. Red dash lines indicate hydrogen bonds between 5α-DHT and the active site residues. The distance from carbonyl (C3) of 5α-DHT to C4N in nicotinamide ring of NADPH or hydroxyl (OH) of Tyr193 in their representative structures is represented in the inserted figures with interatomic distances given in nm. (C) The distance between C3 atom of 5α-DHT and C4N atom of NADPH. (D) The distance between C3 atom of 5α-DHT and OH atom of Tyr193. These distances were measured during 20 ns MD simulation time and the average distance for last 5 ns of the simulation was also presented. WT and C-terminal-deleted PTCRs are colored in green and yellow lines, respectively.

On the other hand, 5α-DHT binding has shown slight changes after 20 ns MD simulation compared with our previous results [15]. In the initial docked structure, 5α-DHT in WT PTCR formed hydrogen bonds with Tyr193, Met234, and Pro284 but these interactions were broken during the simulation because the carbonyl group of 5α-DHT is gradually moved away from these residues and then the hydroxyl group forms hydrogen bonds with Lys238 and Asn287 (Figure 5A). Also, it binds to the active site of WT PTCR through hydrophobic interaction with the residues such as Leu96, Trp229, Met234, Gly235, Pro284, Trp285, Val286, and NADPH (Figure 5A). In case of the binding conformation of 5α-DHT in C-terminal-deleted PTCR, the residues Gln95, Thr140, Glu141, Trp229, Met234, Gly235, and NADPH have formed hydrophobic interaction with 5α-DHT (Figure 5B). Unlike WT PTCR, carbonyl group of 5α-DHT in C-terminal-deleted PTCR makes strong hydrogen bonds with hydrogen atoms of Ser139 and Tyr193 compared with the initial docked conformation. These interactions might contribute to stabilize 5α-DHT binding in the active site of C-terminal-deleted PTCR. Since the hydroxyl group of Tyr193 has been regarded as the proton donor in an electrophilic attack on the carbonyl group of substrate in a reduction reaction [2], hydrogen bond interaction with catalytic Tyr, Tyr193 is especially important for the initiation of the reaction. Although 5α-DHT in WT PTCR binds in a similar fashion with that in C-terminal-deleted PTCR, hydrogen bonds with catalytic residues are only found in C-terminal-deleted PTCR. In addition to, 5α-DHT binding in WT PTCR seems that it somewhat faces toward the C-terminal tail due to hydrophobic interaction with C-terminal tail such as Pro284, Trp285, and Val286.

### Comparison of Distance between 5α-DHT and Tyr193, Mediating Catalytic Reaction in PTCR

As discussed above, C4 position of the nicotinamide ring in NADPH and C3 position of 5α-DHT is a significant factor for hydride transfer reaction. In other words, the carbonyl group of 5α-DHT should be positioned close to the both C4 atom of NADPH and hydroxyl group of Tyr193 to take place the reduction reaction. In our simulation, the distances from 5α-DHT to NADPH or to Tyr193 are relatively short in C-terminal-deleted PTCR compared with WT PTCR (Figure 5A and 5B). The C3 of 5α-DHT in each representative structure is at a distance of 0.48 nm and 0.34 nm from C4 of NADPH in WT PTCR and C-terminal-deleted PTCR, respectively. The distance between C3 of 5α-DHT and the hydroxyl group of Tyr193 is 0.42 nm in WT PTCR and 0.29 nm in C-terminal-deleted PTCR. These distances were monitored throughout the simulation time. First, the distance between carbon C4N in the nicotinamide ring of NADPH and carbon C3 in carbonyl group of 5α-DHT is similar in both simulations until 14 ns but since then it is increased in WT PTCR, while the distance is maintained at 0.4 nm in C-terminal-deleted PTCR (Figure 5C). The average distance during last 5 ns is 0.51 nm and 0.39 nm in WT and C-terminal-deleted PTCRs. Second, the distance between the hydroxyl group of Tyr193 and C3 in the carbonyl group of 5α-DHT is also revealed comparable pattern with above one. The distance in WT PTCR is gradually increased up to 0.8 nm from 10 ns, whereas it is kept around 0.4 nm in C-terminal-deleted PTCR until the end of the simulation (Figure 5D). The average distance for last 5 ns is 0.49 nm and 0.37 nm in WT and C-terminal-deleted PTCR, respectively.

The comparisons of two major distances show that the distances in C-terminal-deleted PTCR are relatively shorter than that of WT during the 20 ns MD simulation. Moreover, this indicates that the binding of 5α-DHT in C-terminal-deleted PTCR makes the protein more favorable condition for initiating the reaction.

### Comparison of WT and C-terminal-deleted PTCRs from an Energetic Perspective

From the each simulation, coulomb and Leonard-Jones potentials between the protein and 5α-DHT were calculated during the entire simulation time. The coulomb potentials are slightly low in C-terminal-deleted PTCR with average potentials of −57.62 kJ/mol while −43.38 kJ/mol for WT (Figure S1). On the contrary, Leonard-Jones potentials are relatively low in WT PTCR with average value of −123.10 kJ/mol and −100.72 kJ/mol for WT and C-terminal-deleted PTCRs, respectively (Figure S1). The calculation is also completed using four repetitive simulations called Rep1 to Rep4 (Table 3). Similarly, the results show that strong coulomb potentials in C-terminal-deleted PTCR while strong Leonard-Jones potentials in WT PTCR. We have also calculated interaction energy estimated as the sum of the van der Waals energy and electrostatic energy using DS. All individual representative structures from the repetitive simulations were used in the calculation and each value was summarized (Table S1). Although the average of interaction energies is not significantly different in both systems with −40.23 kJ/mol and −34.51 kJ/mol for WT and C-terminal-deleted PTCRs, this study reveals that WT PTCR has more stable interactions in terms of van der Waals energy, whereas C-terminal-deleted PTCR has somewhat more stable electrostatic energy. Taken together, these differences might be resulted from that 5α-DHT in WT PTCR forms more hydrophobic interactions with the C-terminal residues while C-terminal-deleted PTCR has more hydrogen bond interaction than WT during the simulation time (Figure S2).

**Table 3:** The calculation of coulomb and Leonard-Jones potentials in the repetitive simulations.

System	WT PTCR		C-terminal-deleted PTCR	
	Coulomb potentials	Leonard-Jones potentials	Coulomb potentials	Leonard-Jones potentials
Rep1	−33.50	−150.04	−50.84	−102.84
Rep2	−51.36	−121.74	−49.88	−114.48
Rep3	−53.13	−128.22	−56.94	−97.35
Rep4	−34.80	−128.82	−55.47	−95.17

Each value indicates the average energy for 20 ns MD simulation and given in kJ/mol.

### The Binding Conformation of 5α-DHT in C-terminal-deleted PTCR Is More Favorable for Catalytic Reaction

**Figure 6:**
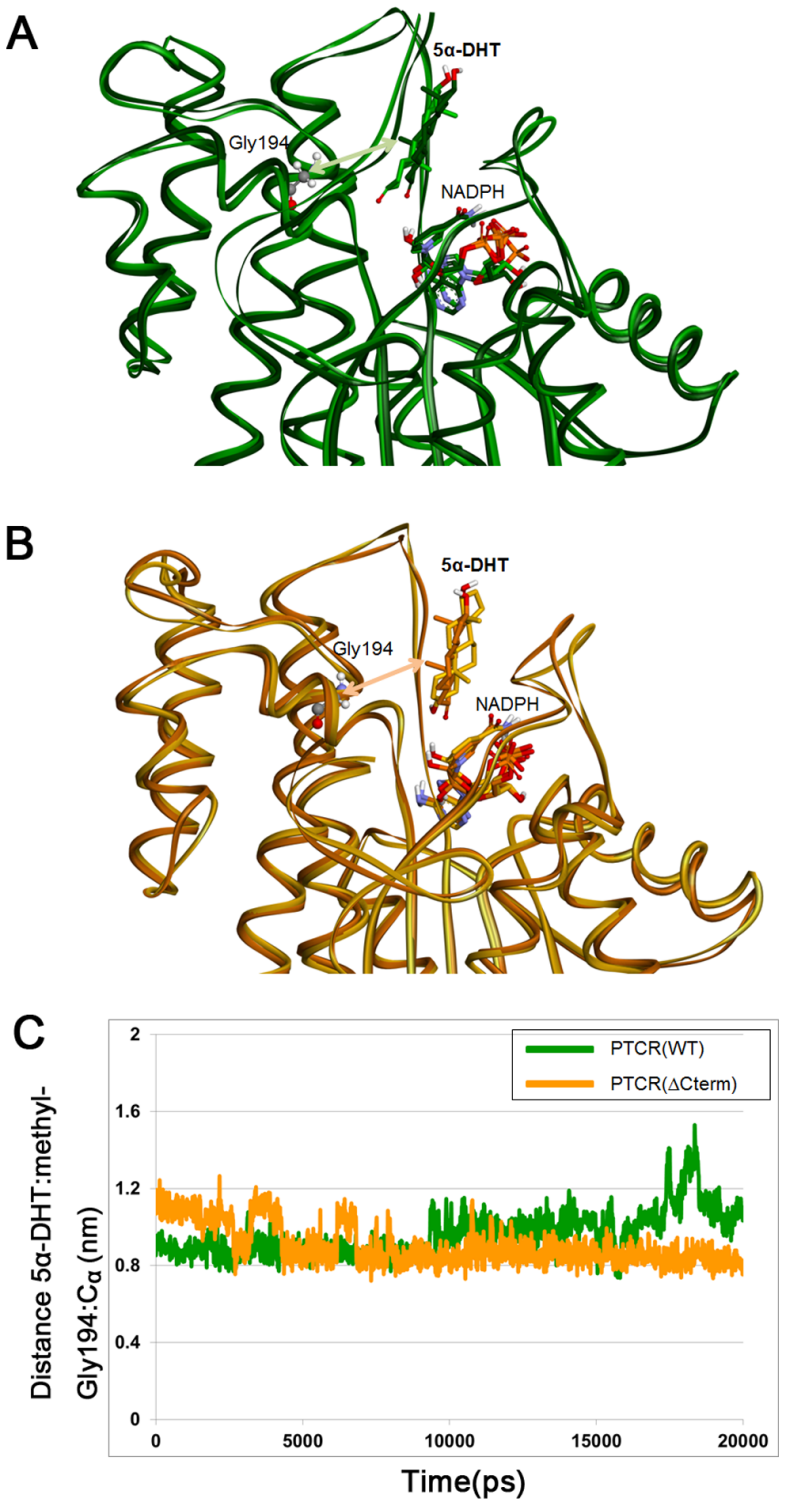
Structural comparison using the extreme configurations obtained from essential dynamics analysis. The configurations taken from the simulation of WT PTCR (A) and C-terminal-deleted PTCR (B) were superimposed based on C_α_ atoms of protein. The configurations showing maximum and minimum movements are displayed as dark and light colors, respectively. 5α-DHT, NADPH, and Gly194 are represented as stick models. The green and orange lines indicate distance between the methyl at C10 position of 5α-DHT and C_α_ atom of Gly194 in WT and C-terminal-deleted PTCRs. (C) The distance between 5α-DHT and Gly194 in WT and C-terminal-deleted PTCRs. The rotation of β-face of 5α-DHT was analyzed by calculating the distance between methyl at C10 position of 5α-DHT and C_α_ atom of Gly194. The distance in WT and C-terminal-deleted PTCRs is shown as green and yellow lines.

**Figure 7:**
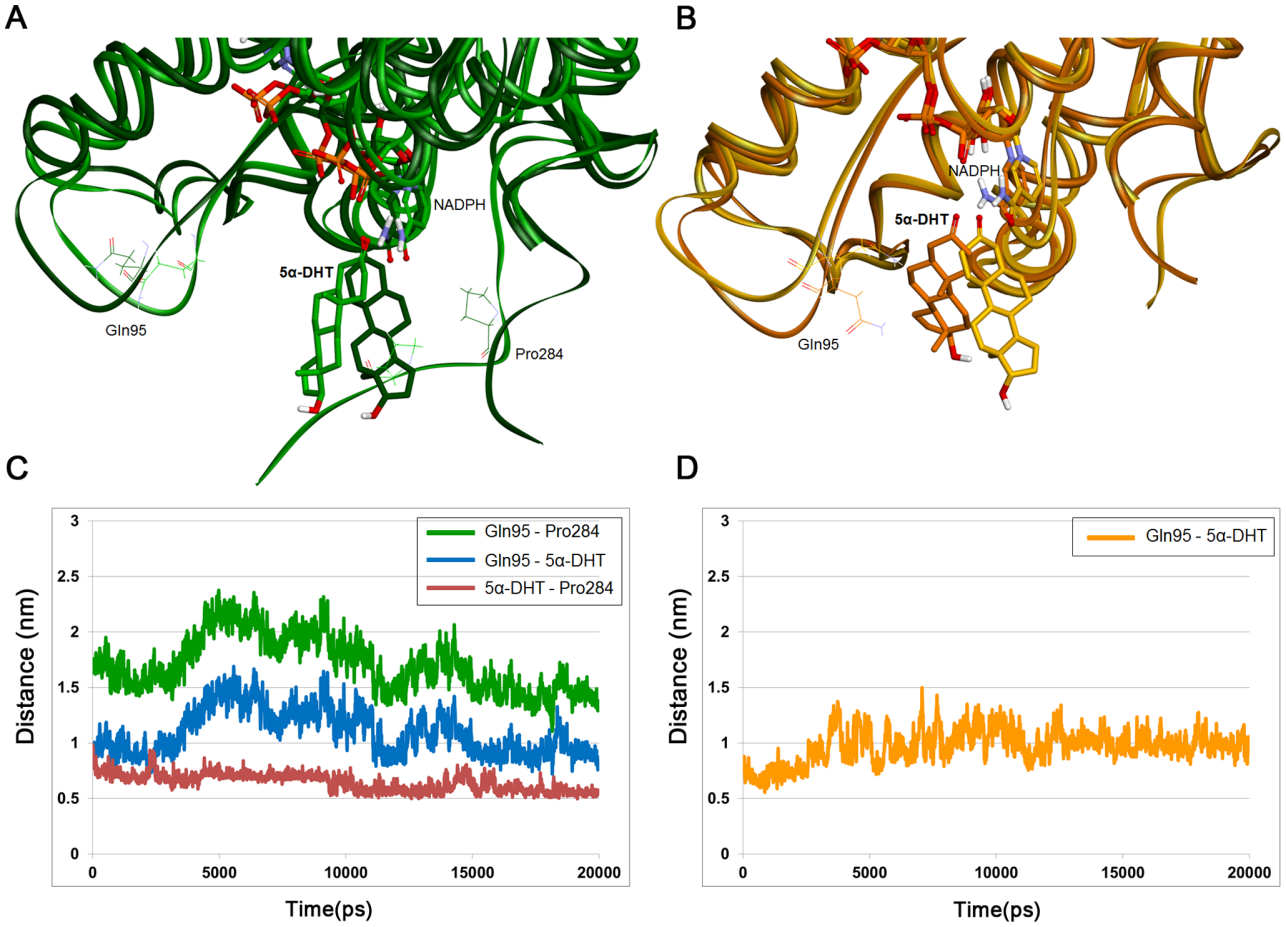
Structural correlation between 5α-DHT binding and C-terminal tail in WT PTCR. Superposition of extreme configurations obtained from essential dynamics analysis for WT PTCR (A) and C-terminal-deleted PTCR (B). The configurations showing maximum and minimum movements are displayed as dark and light colors, respectively. 5α-DHT, NADPH, Gln95, and Pro284 are represented as stick models. (C) The distances between Gln95 and Pro284, Gln95 and 5α-DHT, and 5α-DHT and Pro284 in WT PTCR were calculated during 20 ns simulation time. Each distance is shown as green, blue, and red lines, respectively. (D) The distance between Gln95 and 5α-DHT in C-terminal-deleted PTCR is shown as yellow line.

To further explore the influence of C-terminal deletion on 5α-DHT binding, essential dynamics analysis was performed using MD simulation trajectories of both systems. By calculating eigenvalues and eigenvectors obtained from covariance matrix based on 5α-DHT or protein C_α_ atoms, we identified configurations which showed the largest correlated displacements throughout the simulation time. From the comparison of the extreme configurations, we observed that β-face of 5α-DHT was rotated in the opposite direction in both WT and C-terminal-deleted PTCRs (Figure 6A and 6B). To examine the rotational movement of the β-face of 5α-DHT, the distance between the methyl group at C10 position of 5α-DHT and C_α_ atom of Gly194, facing to the substrate, was considered. Since β-face of 5α-DHT is directed towards the active site for catalytic reaction, the distance should be retained short during the simulation time. The distance between methyl group of 5α-DHT and Gly194 is relatively short in C-terminal-deleted PTCR compared to the WT (Figure 6C). In the simulation of WT PTCR, β-face of 5α-DHT has been rotated and far from Tyr193 about 15 ns due to hydrogen bond interaction with Glu141 and then finally placed on the opposite side of the catalytic site. In case of C-terminal-deleted PTCR, β-face of 5α-DHT was not headed for the active site in the initial stages of the simulation but it slowly turned toward to the catalytic site and was kept properly until the end of the simulation. The comparison of the distance revealed that C-terminal-deleted PTCR retains the right direction of 5α-DHT longer than WT PTCR. Furthermore, superimposition of extreme configurations calculated based on all C_α_ atoms in the protein indicates that hydroxyl tail of 5α-DHT is alienated from active site due to interaction with the residues in C-terminal region in WT PTCR (Figure 7). Changes in distances were considered to confirm the structural correlation between 5α-DHT binding and C-terminal tail. As represented in the figure, C_α_ atoms of Gln95 and Pro284 which were placed in spatially parallel with center of 5α-DHT were used in the distance calculation (Figure 7A and 7B). In WT PTCR, the changes in the distance from Gln95 to 5α-DHT and Pro284 are shown a similar pattern with each other, while the distance between Pro284 and 5α-DHT is maintained at constant value over the simulation (Figure 7C). This indicates that C-terminal tail has formed stable interactions with 5α-DHT and it might restrict the substrate to approach to the catalytic residues. However, there is no significant change in the distance between Gln95 and 5α-DHT in C-terminal-deleted PTCR (Figure 7D). From the essential dynamics analyses we demonstrate that the rotational displacement for β-face of 5α-DHT and structural correlation between 5α-DHT binding and C-terminal tail in WT. These observations are supported by distance calculations and the structural comparisons using extreme configurations.

## Conclusions

In the present study, the kinetic studies using 5α-DHT revealed that deletion of C-terminal tail had significant effects on enzymatic properties of PTCR. While *K*_m_ for 5α-DHT was slightly lower in WT than that of C-terminal-deleted PTCR, reduction rate and catalytic efficiency for 5α-DHT were remarkably increased in C-terminal-deleted PTCR compared to the WT. To find structural differences upon 5α-DHT binding in WT and C-terminal-deleted PTCRs, 20 ns MD simulations for the both complexes were performed and the reasonable explanations for these differences have been discussed in this work. In case of 5α-DHT binding in WT PTCR, it mostly depended on hydrophobic interactions with active site residues and hydrogen bonds with Lys238 and Asn287 were also found. On the contrary, C-terminal-deleted PTCR shows strong hydrogen bond interactions with catalytic residues such as Ser139 and Tyr193. In addition, the distance from 5α-DHT to NADPH or to Tyr193, which is the important distance for hydride transfer reaction, was relatively short in C-terminal-deleted PTCR than that of WT during the whole simulation time.

**Figure 8:**
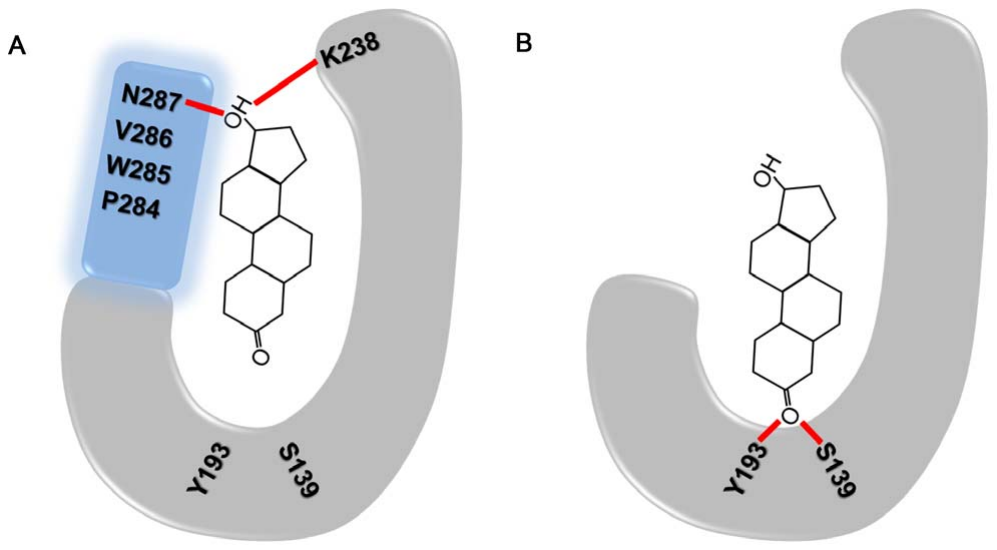
Schematic representation of 5α-DHT binding in WT and C-terminal-deleted PTCRs. (A) The binding modes of 5α-DHT in WT PTCR. (B) Equivalent binding mode in C-terminal-deleted PTCR. These are drawn as diagrams to clearly show the difference in binding conformations between two structures. The protein and C-terminal tail of WT PTCR is illustrated as U-shape (gray) and rectangle (cyan), respectively. The residues involved in the interaction are labeled and red bars indicate hydrogen bonds with 5α-DHT.

From the structural comparisons based on the distance and interaction energy calculations as well as essential dynamics analyses, our results strongly suggest that 5α-DHT binding in C-terminal-deleted PTCR is structurally more favorable for catalytic reaction compared to the WT. Also, these explanations are in accordance with our experimental result that C-terminal-deleted PTCR has higher catalytic efficiency for 5α-DHT than that of WT. On the basis of the overall results obtained from molecular modeling studies, we propose the schematic diagram to easily understand the difference of 5α-DHT binding between WT and C-terminal-deleted PTCRs (Figure 8). Since molecular dynamics studies for explaining enzymatic properties of PTCR have not studied much until recently, we believe that our findings can provide very meaningful information to understand substrate specificity of PTCR and further kinetic properties of enzymes belonging to the SDR superfamily.

## Supporting Information

Figure S1 Interaction energy between the protein and 5α-DHT. (A) The coulomb potentials. (B) Leonard-Jones potentials. These energies were monitored during 20 ns MD simulation time. The energies calculated from WT and C-terminal-deleted PTCRs are given in green and yellow lines, respectively.
(TIFF)

Figure S2 Comparison of the number of intra-hydrogen bonds in WT and C-terminal-deleted PTCRs. The number of hydrogen bonds between the protein and 5α-DHT was monitored during 20 ns simulation time. WT and C-terminal-deleted PTCRs are colored in green and yellow lines, respectively.
(TIFF)

Table S1 Interaction energies between the protein and 5α-DHT. The energies were calculated using representative structure of each repetitive simulation and given in kJ/mol.
(DOCX)
