# Supplementary figures and images for: Functional Mechanism of C-Terminal Tail in the Enzymatic Role of Porcine Testicular Carbonyl Reductase: A Combined Experiment and Molecular Dynamics Simulation Study of the C-Terminal Tail in the Enzymatic Role of PTCR
Source: PLoS One. 2014 Mar 19;9(3):e90712. doi: 10.1371/journal.pone.0090712 (PMC3960098; doi:10.1371/journal.pone.0090712)

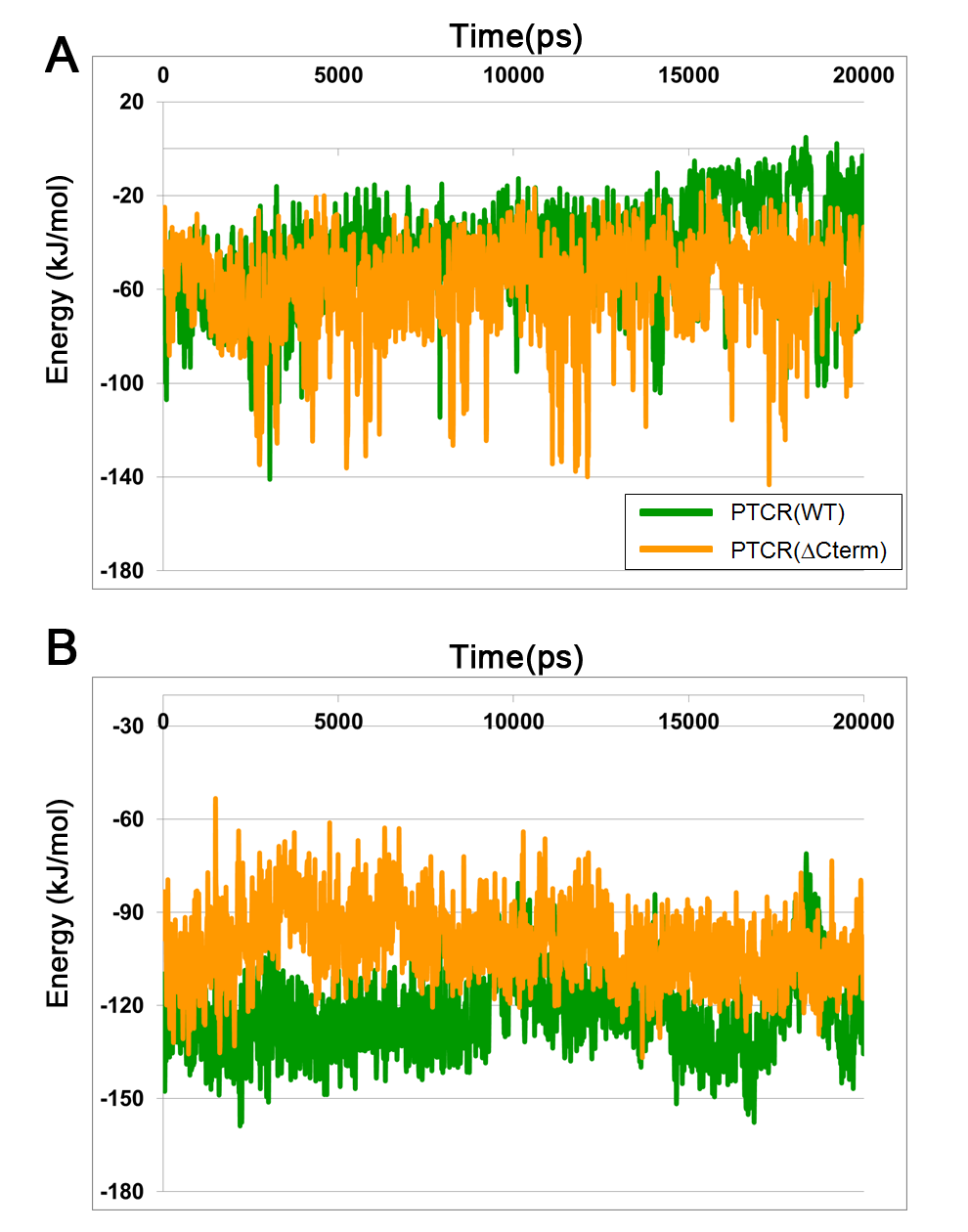

Supplement: Figure S1 — Interaction energy between the protein and 5α-DHT. (A) The coulomb potentials. (B) Leonard-Jones potentials. These energies were monitored during 20 ns MD simulation time. The energies calculated from WT and C-terminal-deleted PTCRs are given in green and yellow lines, respectively. (TIFF) [file pone.0090712.s001.tiff]

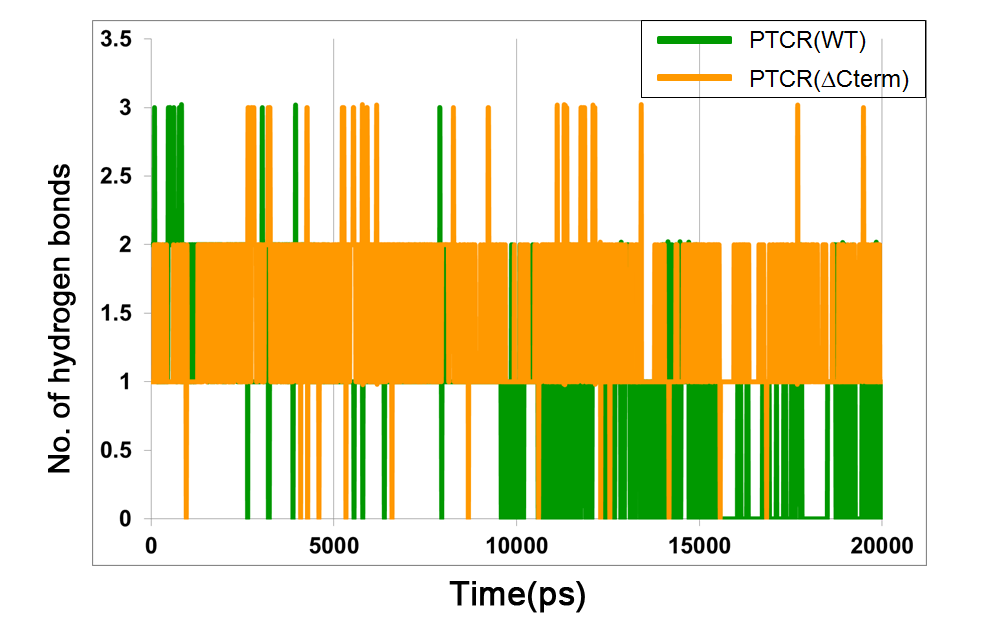

Supplement: Figure S2 — Comparison of the number of intra-hydrogen bonds in WT and C-terminal-deleted PTCRs. The number of hydrogen bonds between the protein and 5α-DHT was monitored during 20 ns simulation time. WT and C-terminal-deleted PTCRs are colored in green and yellow lines, respectively. (TIFF) [file pone.0090712.s002.tiff]
