# Supplementary material for: Functional Mechanism of C-Terminal Tail in the Enzymatic Role of Porcine Testicular Carbonyl Reductase: A Combined Experiment and Molecular Dynamics Simulation Study of the C-Terminal Tail in the Enzymatic Role of PTCR
Source: PLoS One. 2014 Mar 19;9(3):e90712. doi: 10.1371/journal.pone.0090712 (PMC3960098; doi:10.1371/journal.pone.0090712)
Supplement: Table S1 — Interaction energies between the protein and 5α-DHT. The energies were calculated using representative structure of each repetitive simulation and given in kJ/mol. (DOCX) [file pone.0090712.s003.docx]

**Table S1**

|  | WT PTCR | | | C-terminal-deleted PTCR | | |
| --- | --- | --- | --- | --- | --- | --- |
| System | Van der Waals | Electrostatic | Interaction energy | Van der Waals | Electrostatic | Interaction energy |
| Main | -110.00 | -73.47 | -183.47 | -100.83 | -38.70 | -139.53 |
| Rep1 | -139.41 | -32.22 | -171.63 | -96.27 | -59.29 | -155.56 |
| Rep2 | -133.72 | -32.84 | -166.56 | -95.52 | -35.31 | -130.83 |
| Rep3 | -126.61 | -34.43 | -161.04 | -85.69 | -50.00 | -135.69 |
| Rep4 | -116.40 | -42.51 | -158.91 | -103.05 | -57.20 | -160.25 |
| Average | -125.23 | -43.10 | -168.32 | -96.27 | -48.10 | -144.37 |
